# Functional parameters of spermatozoa obtained by a new selection device

**DOI:** 10.1002/2211-5463.70073

**Published:** 2025-07-14

**Authors:** Julio C. Chávez, Paulina Torres, Gabriela Carrasquel‐Martínez, María G. Figueroa‐Méndez, Diana Flores, Lina Villar, Israel Maldonado, Marcela B. Trevino, Claudia Treviño

**Affiliations:** ^1^ Departamento de Genética del Desarrollo y Fisiología Molecular, Instituto de Biotecnología (IBT) Universidad Nacional Autónoma de México (UNAM) Cuernavaca Mexico; ^2^ CITMER, Reproductive Medicine México City Mexico; ^3^ Science Department, School of Pure and Applied Science Florida SouthWestern (FSW) State College Fort Myers FL USA

**Keywords:** parameters, separation, sperm, validation

## Abstract

The success of assisted reproduction techniques (ARTs) is based on the selection of gametes with optimal characteristics. This is critical for male gametes, since among the large number of cells present in semen, many may have unnoticeable DNA damage and compromised membrane integrity, among other alterations. In this work, we examined the use of the LensHooke CA0™ device (CA0 chamber) as a promising sperm separation method for ARTs, analyzing both normozoospermic and teratozoospermic samples. Additionally, we compared fertilization rates with IVF and ICSI procedures using Teratozoospermic samples. As reference for comparison, we used the current standard for sperm selection, density gradient centrifugation. Using CA0 chambers and DGC, we obtained comparable sperm recovery numbers, membrane potential (in Normozoospermic samples) and motility parameters. The progesterone‐induced intracellular Ca^2+^ increase was only slightly greater in sperm selected with DGC compared to CA0 chambers. Finally, no differences were observed in IVF and ICSI fertilization rates between Tz sperm separated with DGC and CA0 chambers. Overall, we conclude that the quantity and quality of sperm selected with CA0 chambers is comparable to that obtained with DGC, without compromising reproducibility. Importantly, CA0 chambers offer key practical and methodological advantages, resulting in a faster, simpler and more affordable sperm selection method.

## Graphical abstract

This study compares density gradient centrifugation and the LensHooke CA0™ device for sperm separation. DGC requires multiple steps, specialized equipment, and technical expertise. In contrast, the CA0 device offers key practical and methodological advantages, resulting in a faster, simpler, and more affordable sperm selection method.

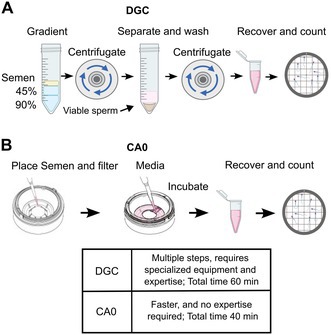

**Abbreviations**
[Ca^2+^]_i_
: intracellular calcium concentration
ARTs: assisted reproduction techniques
CA0: LensHooke CA0™ device
CASA: computer assisted sperm analysis
CITMER: Centro de Innovación Tecnológica y Medicina Reproductiva
DFI: DNA fragmentation index
DGC: density gradient centrifugation
DiSC_3_(5) dye: 3,3′‐Dipropylthiadicarbocyanine Iodide
Em: membrane potential
FDA: U.S. Food and Drug Administration
FSC: forward scatter
GnRH: gonadotropin‐releasing hormone
G‐TL: Oocyte GlobalTotal culture media
hCG: recombinant chorionic gonadotropin
HTF: human tubal fluid medium
ICSI: intracytoplasmic sperm injection
IUI: intrauterine insemination
IVF: *in vitro* fertilization
MACS: magnetic‐activated cell sorting
MAD: median absolute deviation
Nz: normozoospermic
P4: progesterone
ROS: reactive oxygen species
SSC: side‐scatter
STR: straightness
Tz: teratozoospermic
Val: valinomycin
VCL: curvilinear velocity
WHO: World Health Organization

According to the World Health Organization (WHO), around 17.5% of the adult population experiences infertility [1] worldwide. In Mexico, one of every six couples who wish to have a child experiences conceiving problems, which represents around 16% of all couples [2, 3]. There are multiple causes for this, including genetic problems, certain diseases, and even lifestyle or habits, such as smoking. Overall, half of the cases are due to female factors and half to male factors. As a result, a growing number of couples rely on fertility clinics for assisted reproduction techniques (ARTs). About 55% of these couples are successful and become parents [4, 5]. A critical aspect that determines the success of fertilization is the selection of the proper ART, namely, *in vitro fertilization* (IVF) or intracytoplasmic sperm injection (ICSI).

Focusing on the male factor, the viable sperm separation method is pivotal since cell quality is key to the success of fertilization, and it also influences the health of the offspring. Therefore, important efforts have been made in the development of separation methods that favor the abundance of cells with good motility, normal morphology, and DNA integrity [6]. For example, the swim‐up method is among the oldest, and while it is functional, simple, and inexpensive, it is not as efficient in terms of the number of cells recovered compared to other selection methods [7]. Therefore, the use of swim‐up is recommended for samples with a high cell count, which is not always the case for patients in fertility clinics [6, 8]. Methods using either glass wool filtration or discontinuous Percoll gradient centrifugation have been developed in the past. At that time, both methods represented good selection procedures in terms of sperm motility, although they were not as efficient in patients with asthenozoospermia [9]. However, in 1996, Percoll was withdrawn from use in fertility clinics due to the risk of contamination with endotoxins [6]. Since then, alternative commercial compounds have emerged to perform density gradient centrifugation (DGC) and enable the selection of viable sperm without the risk of contamination [10, 11, 12]. Further, in order to avoid the use of gradient components that may be detrimental to ARTs, for example, by producing inflammatory side effects such as an allergic response, some methods that did not involve sample centrifugation were also developed and tested [13]. One of them is the migration‐sedimentation procedure, which is a combination of swim‐up with a sedimentation step [14].

Additionally, efforts have been made to study the effects that the different separation methods have on sperm at the molecular and functional levels. For example, it was previously described that sperm separated by DGC showed spontaneous intracellular Ca^2+^ ([Ca^2+^]_i_) oscillations of greater frequency and amplitude than sperm selected by swim‐up. Likewise, sperm obtained by DGC displayed a higher percentage of hyperactivation and increased protein tyrosine phosphorylation levels, indicating a ‘better capacitated’ state compared to sperm separated by swim‐up [15]. Other approaches for sperm selection to select the best sperm involve the development of microfluidic chambers [16, 17]. While various designs have been created, the goal is the same, that is, to achieve separation of the best sperm possible for its use in ARTs. In fact, several reports indicate that the use of microfluidic chambers results in the enrichment of sperm with high motility, good morphology, and a significant reduction in the percentage of sperm with DNA damage [16, 17, 18, 19, 20]. However, the use of these chambers has so far been limited to research purposes, as in fact they have not yet been tested in fertility clinics; thus, their advantages for ARTs are only now starting to be explored [18, 21, 22].

Additionally, there are some sperm selection strategies for ICSI. Some of them include the combination of DGC/Zeta potential [23, 24]. Zeta potential is a selection procedure based on cellular characteristics of spermatozoa such as surface electrical charge (electrokinetic potential) to isolate a normal sperm subpopulation with intact chromatin. The design of the Zeta method came from observing sperm adhering to the surface of glass slides when the culture medium was not supplemented with serum or albumin protein [25]. Mature sperm possess an electric charge of −16 to −20 mV, which decreases with capacitation [25]. Esfahani *et al*. demonstrated that the rates of fertilization, implantation, and pregnancy after the Zeta method were significantly higher than routine sperm preparation procedure in men candidates for ICSI [23].

Another sperm selection procedure consists in the separation of apoptotic from non‐apoptotic sperm, termed magnetic‐activated cell sorting (MACS) [26, 27, 28]. MACS principle is based on conjugated annexin V with magnetic microspheres exposed to a magnetic field in an affinity column. Apoptotic sperm externalizes phospholipid phosphatidylserine, which has a high affinity for annexin V, and therefore they will be bound to the affinity column [26]. This technique efficiently reduces sperm DNA fragmentation levels [27] and improves pregnancy rates [28]. A combination between MACS and DGC has been employed, where some reports compared for instance MACS‐DGC vs. DGC‐MACS for sperm separation [29]. Tavalaee and co authors concluded that although no big differences were observed regarding DNA integrity, chromatin maturity and sperm morphology between the combinations MACS‐DGC vs. DGC‐MACS, they recommended the MACS followed to DGC in order to lower caspase‐positive sperm, since the mentioned combination separates the apoptotic sperm rather than annexin positive ones induced by capacitation [30].

Additional approaches for sperm selection involve the development of microfluidic chambers [16, 17]. While various designs have been created, the goal is the same, that is, to achieve separation of the best sperm possible for its use in ARTs. In fact, several reports indicate that the use of microfluidic chambers results in the enrichment of sperm with high motility, good morphology, and a significant reduction in the percentage of sperm with DNA damage [16, 17, 18, 19, 20]. However, the use of these chambers has so far been limited to research purposes, as in fact they have not yet been tested in fertility clinics; thus, their advantages for ARTs are only now starting to be explored [18, 21, 22].

In this work, we explored a new method of sperm separation using a LensHooke CA0™ device (CA0), which was recently tested and compared with DGC, but only in terms of motility parameters, concentration, morphology, motion kinematics, DNA fragmentation index (DFI), and the rate of acrosome‐reacted sperm (AR) [31]. As suggested in the 6th edition of the *Laboratory Manual for the Examination and Processing of Human Semen* [32], deeper insights relating to human sperm competence may be gained by performing functional tests related to cellular and molecular sperm physiology. Accordingly, in addition to parameters measured by [31], we evaluated the [Ca^2+^]_i_ response to progesterone (P4) and measured the resting membrane potential (Em) of sperm separated by DGC and CA0 from normozoospermic (Nz) donors and teratozoospermic (Tz) patients. Evaluating these parameters is particularly relevant, as there is evidence of a positive correlation existing between fertility and both a hyperpolarized Em (more negative membrane potential values) and the magnitude of the P4‐induced [Ca^2+^]_i_. Indeed, subfertile men have shown smaller responses to P4 compared to men with normal fertility [33], while a positive correlation has been observed between Em hyperpolarization and fertilizing ability for IVF procedures in particular [34, 35, 36]. In fact, the use of Em values as predictors of fertility has been proposed [37]. Finally, we evaluated the fertility rate of sperm obtained by CA0 and DGC using IVF and ICSI methodologies. The evaluation of functional parameters that predict fertilization rates, such as resting Em values and the P4 response, combined with the measurement of the corresponding IVF and ICSI success rates performed in this work, constitute a complete and thorough comparison of the two separations methods. This enables us to validate the use of CA0 as a suitable alternative sperm selection method for ARTs, as it not only eliminates the potentially harmful use of centrifugation and its associated media, but is also faster and simpler than DGC, while being equally reliable.

## Materials and methods

### Ethics and research facilities

This work was carried out jointly in Mexico by the Institute of Biotechnology (UNAM) and CITMER. The protocols employed at the former were approved by the Ethics Committee (Institute of Biotechnology, UNAM), registered under code CF‐2023‐I‐231, project: 450. CITMER protocols were approved by the Internal Review Board, with the number CE‐23‐103 and Registration number NCT06545318, certifying that the study was performed in accordance with the ethical standards as laid down in the 1964 Declaration of Helsinki and its later amendments or comparable ethical standards. The CA0 device has already been tested and approved by several institutions and now is even out for sale. However, since we are studying human cells and samples, we registered this study in clinicaltrials.gov, with the number NCT059191.

CA0 sperm separation device is commercially available approved by U.S. Food and Drug Administration (FDA) No. 0910‐0120K221810.

Written informed consent was obtained from all sperm donors and patients. For the latter, we used only the sample remaining after their medical procedures were concluded.

Normozoospermic (Nz, *n* = 12) and teratozoospermic (Tz, *n* = 46) samples were analyzed at UNAM and CITMER, respectively. Therefore, Em and intracellular Ca^2+^ measurements were performed using the equipment available at each facility, as indicated in the corresponding sections below.

### Materials

The following reagents were obtained from InvitroCare (Frederick, MD, USA): HTF‐HEPES culture medium (NaCl, KCl, MgSO_4_·7H_2_O, KH_2_PO_4_, CaCl_2_·2H_2_O, NaHCO_3_, d‐glucose, sodium pyruvate, sodium lactate, alanyl Glutamine, EDTA tetrasodium salt, gentamicin, phenol red, and HEPES sodium salt, supplemented with 10% human serum albumin pH 7.0 to 7.6; Cat. 2002‐5); human serum albumin (Cat. 2101); upper layer (45%; Cat. 2221); and lower layer (90%; Cat. 2222) gradient media. Oocyte GlobalTotal (G‐TL) culture media (bicarbonate buffered medium containing hyaluronan and human serum albumin, Cat. 10145) was acquired from Vitrolife (Gothenburg, Sweden). Recombinant follicle‐stimulating hormones Rekovelle and highly purified urinary FSH Merapur were obtained from Ferring (St. Prex, Switzerland). Follicle‐stimulating hormone Gonal‐F and human recombinant chorionic gonadotropin (hCG) Ovidrelle were obtained from Merck Serono (Aubonne, Switzerland). Diff‐Quick stain kit (Cat. CTDQ‐250‐003) was acquired from Kubus (Madrid, Spain). Fluo‐3‐AM dye (Cat. 21010) was purchased from AAT Bioquest (Pleasanton, CA, USA). DiSC_3_(5) dye and valinomycin (Cat. D‐306 and V‐1644, respectively) were obtained from ThermoFisher (Nuevo León, Mexico). Ionomycin (Cat. I‐700) was obtained from Alomone (Jerusalem, Israel). Progesterone (Cat. P‐8783) and MnCl_2_ (Cat. M‐3634) were obtained from Sigma‐Aldrich (St. Louis, MO, USA). LensHooke CA0 sperm separation devices were obtained from Bonraybio Co., LTD (Taiwan, Republic of China).

### Sperm preparation

This study included sperm samples from normozoospermic (Nz) donors (ages 18–35) that met the parameters established by the 2010 WHO Laboratory Manual for the Examination and Processing of Human Semen [38] and from teratozoospermic (Tz, characterized by having less than 4% normal sperm morphology) patients (ages 23–60) collected by the Fertility Clinic ‘Centro de Innovación Tecnológica y Medicina Reproductiva’ (CITMER, Mexico City). All patients included in the studies were selected taking into consideration a sperm concentration ≥ 16 × 10^6^ million sperm·mL^−1^. This allowed us to have enough leftover samples to be able to perform the cytometry test.

**Fig. 1:**
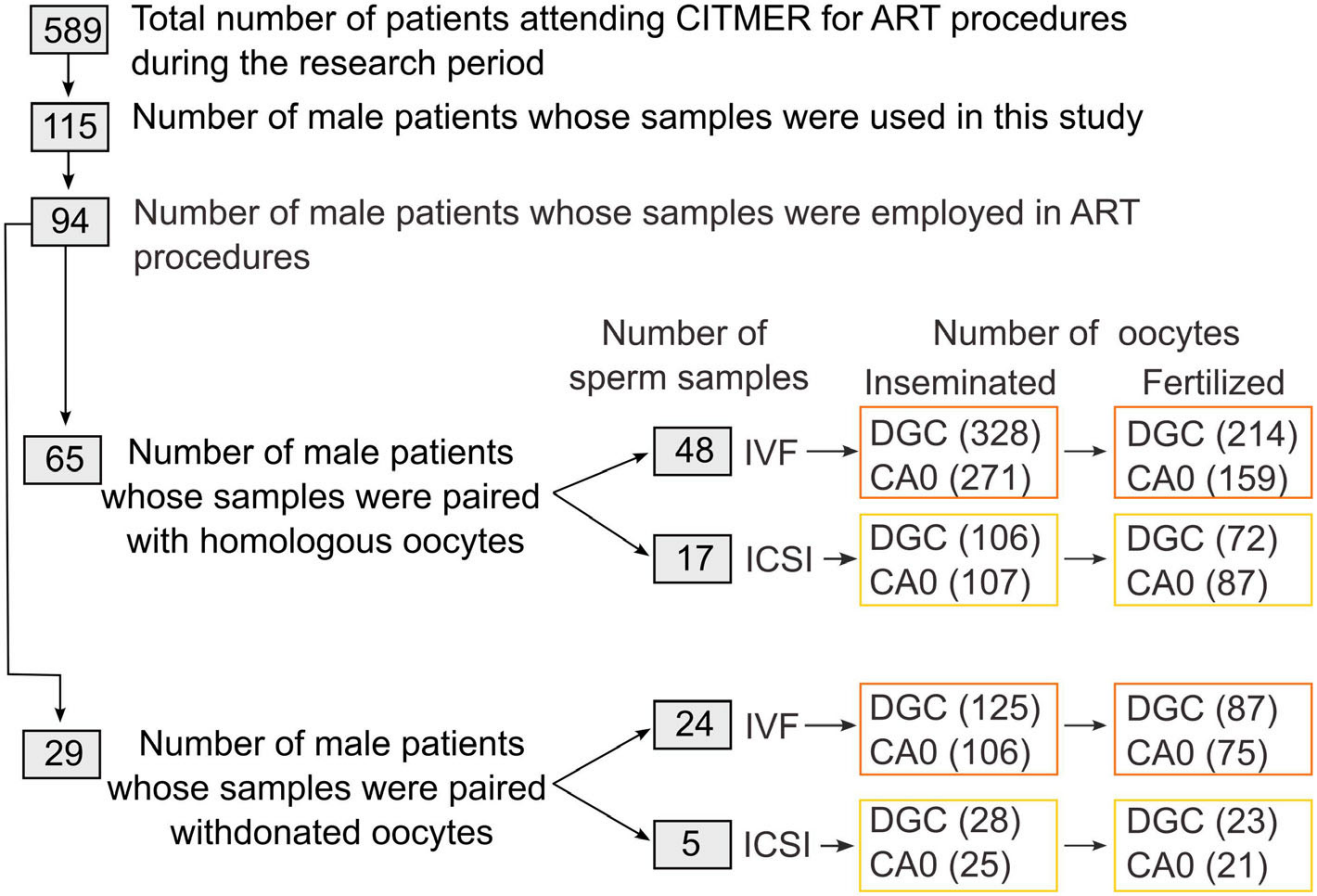
Experimental scheme of the sperm and oocyte samples used in this study. The number of male patients/sperm samples, number and origin of oocytes, types of ART procedure, and fertilization success numbers are indicated. Sperm samples were separated using either density gradient centrifugation (DGC) or CA0 chambers (CA0).

Sperm morphology evaluation was carried out using the Diff‐Quik stain kit, following the criteria in the aforementioned 2010 WHO Manual. Nz donors had at least 2 days of sexual abstinence. At least 2 mL of semen was obtained for each Nz and Tz sample. Samples were liquefied for 30 min at 37 °C with 5% CO_2_ and immediately divided into two equal parts for cell separation by either DGC or CA0. Cell counts were performed with a Makler chamber (Microptic, Spain) for each semen sample, both before and after sperm separation. For sperm dilution, commercial HTF‐HEPES medium was used (Fig. 1).

### Sperm separation by density gradient centrifugation (DGC)

**Fig. 2:**
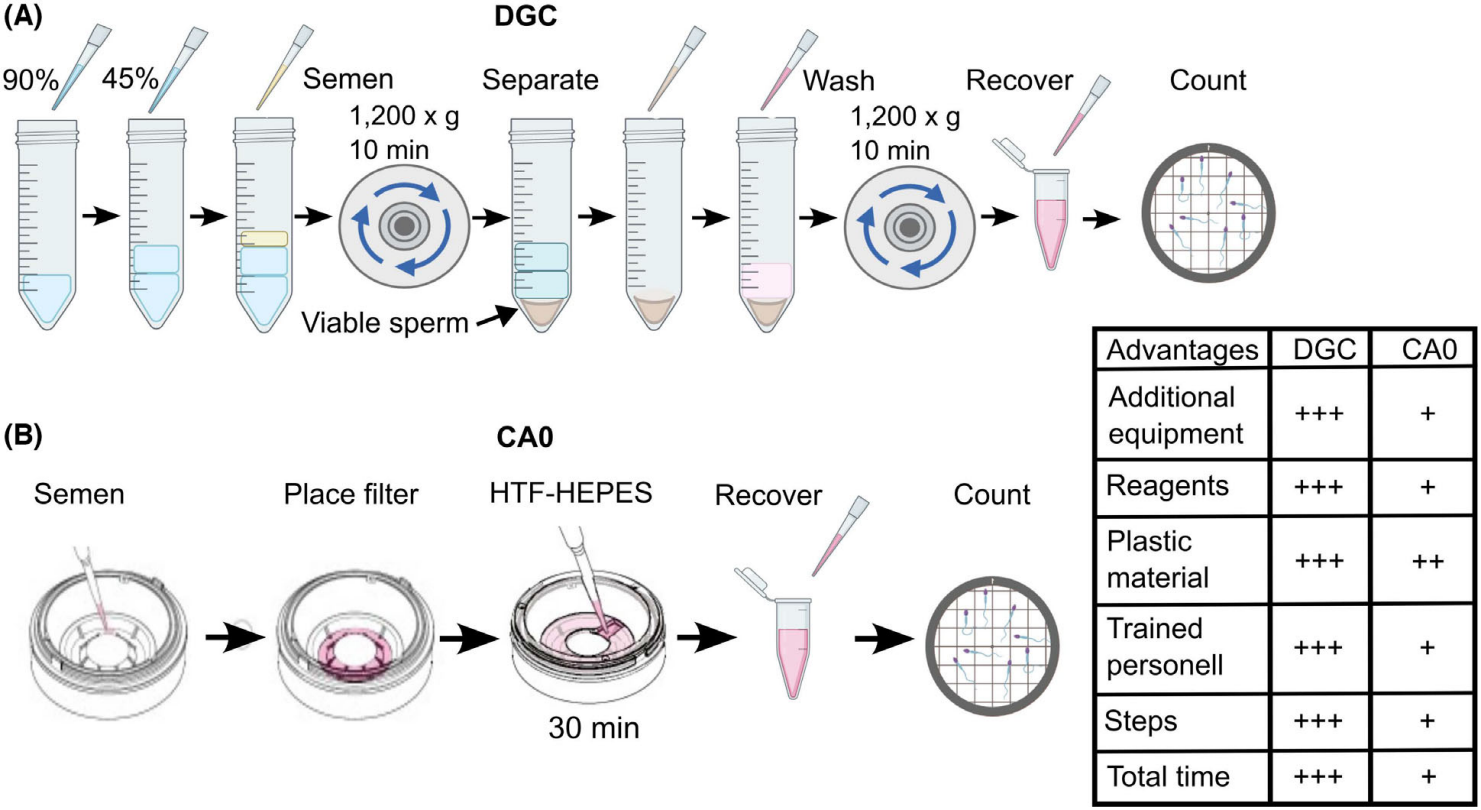
Schematic diagram of sperm separation procedures. (A) Density Gradient Centrifugation (DGC): A density gradient is formed by placing 1 mL of lower phase (90%) medium followed by 1 mL of upper phase (45%) medium, and a 1‐mL semen sample is deposited on top. The gradient is centrifuged at 1200 ***g*** for 10 min, and after removing the supernatant, the cell pellet is resuspended in 1 mL of HTF‐HEPES medium and then centrifuged for 10 min at 1200 ***g***. The resuspended cell pellet containing motile/viable sperm is recovered and an aliquot is used for the cell count. (B) CA0 Chambers (CA0): A 1‐mL semen sample is placed in the base of a CA0 chamber, the filter that selects the cells is placed above the base. 900 μL of medium is placed inside the chamber, and the chamber is covered with the lid. After 30 min, the medium containing the selected cells is recovered and an aliquot is used for the cell count. Partially taken from LensHooke user manual (https://lenshooke.com/support.php?cat=38&pid=202).

A gradient is formed in 15 mL conical tubes as follows: 1 mL of lower gradient (90%) medium is placed at the bottom, 1 mL of upper (45%) gradient medium is placed on top of the bottom layer, and 1 mL of semen sample is layered above the gradient. Tubes are centrifuged for 10 min at 1200 ***g*** at room temperature. The pellet containing the viable cells is separated, resuspended in 1 mL of HTF‐HEPES medium, and centrifuged at room temperature for 10 min at 1200 ***g*** to remove traces of gradient medium. The supernatant is then removed, 500 μL of HTF‐HEPES medium are added to the sperm pellet, carefully mixed, and cells are counted (Fig. 2A).

### Sperm separation by LensHooke CA0™ device (CA0 chamber)

The mechanism of the CA0 chamber (Bonraybio, Taichung, Taiwán) is based on the physiological principle of sperm motility; it selects motile sperm that can self‐propel through the micropores of a membrane, separating them from immotile sperm, other cell types, and debris present in seminal plasma. The CA0 chamber is made up of three components: a lower chamber, an upper chamber, and a cover. The upper chamber has a built‐in polycarbonate membrane filter and also has a retrieval port for recovering motile sperm [31]. The procedure suggested by the manufacturer of the CA0 chambers was followed. Briefly, the CA0 device consists of three parts, namely a base, an intermediate part with the separation filter, and a lid. One mL of semen is placed in the base, then the intermediate part is placed on the base, taking care to match the notches that assemble both parts. Subsequently, 900 μL of HTF‐HEPES medium is placed in the intermediate part, the lid is placed, and the device is incubated at 37 °C for 30 min. Subsequently, a 500‐μL aliquot is recovered and cells are counted (Fig. 2B).

### Computer‐assisted sperm analysis (CASA)

Motility parameters were evaluated for semen samples and for sperm separated by DGC or CA0. For Nz samples, these parameters were evaluated using computer‐assisted sperm analysis equipment coupled to a Nikon Eclipse Ci‐L upright microscope with a thermo‐adjustable stage and sperm class analyzer 5 software (SCA Version 5.4 Microptic, Spain), equipped with a Basler Ace camera (Germany) with a 10× objective. Velocity thresholds were set at < 10 μm·s^−1^ (slow), 10–35 μm·s^−1^ (medium), and > 35 μm·s^−1^ (fast), as per system defaults. Sperm motility was assessed based on curvilinear velocity (VCL), and sperm samples were classified as either progressive (STR > 80%), non‐progressive (80% > STR > 0%), or immotile (STR = 0%). Cell motility was performed by depositing 10 μL of sample in spermtrack‐20 sperm‐counting chambers (Projectes i serveis, Valencia, Spain). At least 300 cells from each sample were evaluated by acquiring images at 50 Hz for 3 s. For Tz samples, motility was visually assessed in a microscope Olympus CX43 with a 20× objective; at least 100 cells were counted per sample.

### Membrane potential measurements

**Fig. 3:**
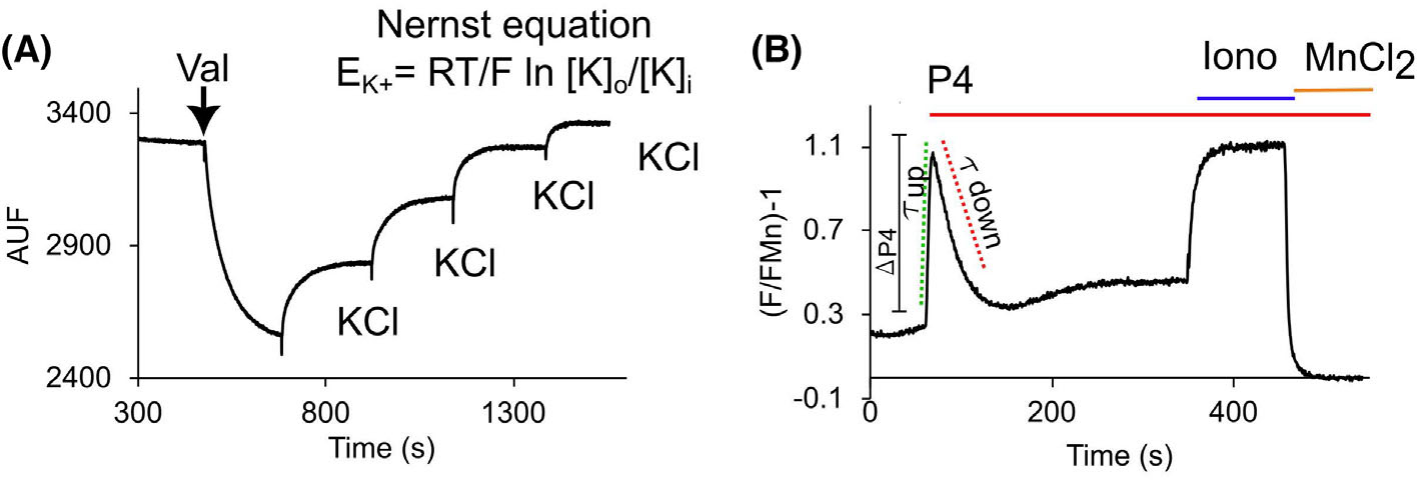
Fluorescence measurements of membrane potential and intracellular Ca^2+^. (A) Membrane potential (Em) calibration: Representative DiSC_3_(5) fluorescence trace showing the fluorescence change after the addition of the K^+^ ionophore valinomycin (Val), followed by several additions of known concentrations of KCl. Fluorescence values are correlated with theoretical values of the K^+^ equilibrium potential using the Nernst equation, where R = universal ideal gas constant (8.314 J K^−1^·mol^−1^); T = temperature in Kelvins; F = Faraday constant (96485.33 °C·mol^−1^); ln = natural log; [K]_o_ = outside K^+^ concentration; [K]_i_ = inside K^+^ concentration. (B) Intracellular Ca^2+^ measurement: Representative Fluo‐3 fluorescence trace showing the fluorescence change following the addition of 3 μm Progesterone (P4; solid red line). As controls, 10 μm Ca^2+^ ionophore ionomycin (Iono, blue solid line) is added followed by 5 mm MnCl_2_ (yellow solid line). The magnitude of the P4 response (ΔP4), the half‐time (τ up) to reach the maximum P4 response (green dotted line), and the half‐time (τ down) to reach maximum recovery (red dotted line) are calculated using Origin 2023.

Em measurements were performed at room temperature. Forward scatter (FSC) and side‐scatter (SSC) fluorescence data were collected for each samples. NZ samples were adjusted with HTF medium to a concentration of 10 × 10^6^ cells·mL^−1^, incubated for 5 min at 37 °C with the potential‐sensitive dye DiSC_3_(5) (1 μm), and placed in a 1 mL flat‐bottomed cylindrical glass cuvette. Em measurements were performed using an Aminco SLM spectrofluorometer operated with olis software. A 640 nm LED lamp was used as the illumination source, and emission was adjusted to 640 nm with a monochromator. During recordings, the temperature was maintained at 37 °C with a circulating water bath. Calibration for Em determination was performed by adding 1 μm valinomycin, followed by sequential additions of either a 0.5 m or a 1 m KCl stock solution, to achieve sequential KCl final concentrations (mm): 7.5, 12.5, 22.5, and 42.5 (taking into consideration that HTF medium contains 5 mm KCl) (Fig. 3A). Theoretical Em values were determined using the Nernst equation, assuming that the intracellular K^+^ concentration ([K^+^]_i_) is 120 mm, as determined by Babcock (1983) in bovine sperm [39]. Cell resting Em values were obtained by interpolating the theoretical values with arbitrary fluorescence values. Em measurements in Tz samples were performed with a BD Accuri C6 Plus Flow cytometer (Becton Dickinson, San Jose, CA, USA) which was retrofitted to perform time‐lapse fluorescence acquisitions. For a detailed description of the methodology and use of the aforementioned cytometer, see the work of Matamoros‐Volante *et al*. [37]. Fluorescence was acquired in the cytometer at a flow rate of 14 μL·min^−1^. Threshold levels for FSC and SSC were set to exclude cellular debris, and a two‐dimensional density plot of FSC height (FSC‐H) versus FSC area (FSC‐A) was used to eliminate cellular aggregates from the analysis. DiSC_3_(5) was excited with a 640 nm laser, and its emission was detected in the FL4‐channel set with a 670 nm LP filter. Prior to fluorescence acquisition, cells were stained with propidium iodide (PI) to exclude dead cells. Only single and viable cells were included in subsequent analyses.

### Intracellular Ca^2+^ ([Ca^2+^]_i_) measurements

An aliquot of each sample is adjusted to 10 × 10^6^ cells·mL^−1^ with HTF medium, then loaded with 2 μm Fluo‐3‐AM dye for 30 min at 37 °C and 5% CO_2_. In the case of Nz samples, [Ca^2+^]_i_ measurements were performed by spectrofluorometry using an Aminco SLM spectrofluorometer operated by olis software (Bogart, GA, USA) with a 490 nm blue LED lamp as the illumination source, and emission adjusted to 515 nm with a monochromator. Cells are placed in a 0.5 mL flat‐bottomed cylindrical glass cuvette and population fluorescence is acquired before and after the addition of 3 μm P4. At the end of the P4 response, 5 μm ionomycin were added, followed by 5 mm MnCl_2_ to obtain the maximum and minimal fluorescence signal, respectively. During recordings, the cuvette was maintained at 37 °C using a circulating water bath. Similar to Em measurements, [Ca^2+^]_i_ determinations for Tz samples were performed with the retrofitted flow cytometer as described above, except that to detect Fluo‐3 fluorescence, the 488 nm laser was used as the excitation source and emission signals were detected in the FL‐1 channel with a 533/30 nm filter [37]. [Ca^2+^]_i_ data were normalized by considering the minimal fluorescence value obtained after the addition of MnCl_2_ equivalent to zero. For each sample, the maximum response to P4 was obtained. Also, ΔP4 was calculated, which means the difference between the maximal fluorescence response – basal fluorescence. Finally, we determined the τ up and τ down, which represent the speed to reach the maximum response and to return to basal levels, respectively (Fig. 3B).

### ART procedures

ART cycles were performed on both homologous oocytes and donated oocytes (recipients), which were inseminated by either IVF or ICSI depending on the quality and maturation of the gametes. For this study, only cycles with 4 or more oocytes retrieved were considered. Women underwent regular ovarian stimulation using either a protocol according to CITMER's standard operating procedures involving a gonadotropin‐releasing hormone (GnRH) agonist or antagonist, combined with recombinant follicle‐stimulating hormones indicated in the Materials section. The administration of hCG for final follicular maturation was performed once ≥ 3 preovulatory follicles (16–22 mm in diameter) were observed and Estradiol levels per preovulatory follicle were > 200 pg·mL^−1^. Oocyte retrieval occurred 36 h after hCG administration by transvaginal ultrasound‐guided aspiration. The collected oocytes in HTF‐HEPES medium supplemented with human serum albumin, were then placed in G‐TL culture medium. Conventional IVF and ICSI procedures were performed 3–6 h after oocyte collection as recommended in [40]. Each cohort of sibling oocytes per cycle was divided into two groups for insemination with the partner's capacitated sperm. Half of the oocytes were inseminated with sperm recovered from DCG, and the other half with sperm recovered from CA0. Inseminated oocytes were placed in G‐TL culture medium at 37 °C, 8% CO_2_, 20% O_2_ for 17 to 20 h and were considered fertilized by the presence of 2 pronuclei and two polar bodies [41] (Fig. 1). Fertilization rates were calculated by dividing the number of zygotes obtained by the number of mature oocytes employed (number of zygotes/total number of mature oocytes inseminated). The 0 PN and 1 PB, 0 PN, and 2 PB oocytes were considered as not fertilized. The 3 PN and 2 PB oocytes were considered as triploid and discarded. Given that this study is subject to a confidentiality agreement with CITMER, only fertilization rate data was available to assess ART success. The oocytes with diminished and dark cytoplasm were considered degenerated post ICSI. For Tz samples only, ART cycles were performed on both fresh homologous and donated oocytes (recipients), which were inseminated by IVF or ICSI (MII oocytes).

### Sperm sample selection criteria

For comparison purposes, we employed the same sample volume (1 mL) for each separation method; therefore, only semen samples with a volume of at least 2 mL of semen were included. In addition, we excluded samples from patients with severe oligozoospermia, and we exclusively used fresh semen samples, rather than frozen ones.

### Statistical analysis

Since semen results are paired between DGC and CA0 separation procedures, the statistical test used for data analysis is Wilcoxon signed rank test for paired data (two‐sided), using kyplot 6.0 software (KyensLab, Tokyo, Japan). The data are presented as the median value ± the median absolute deviation (MAD). As indicated in each plot, statistically significant differences were considered when *P* ≤ 0.05, *P* ≤ 0.01 or *P* ≤ 0.001.

## Results

**Fig. 4:**
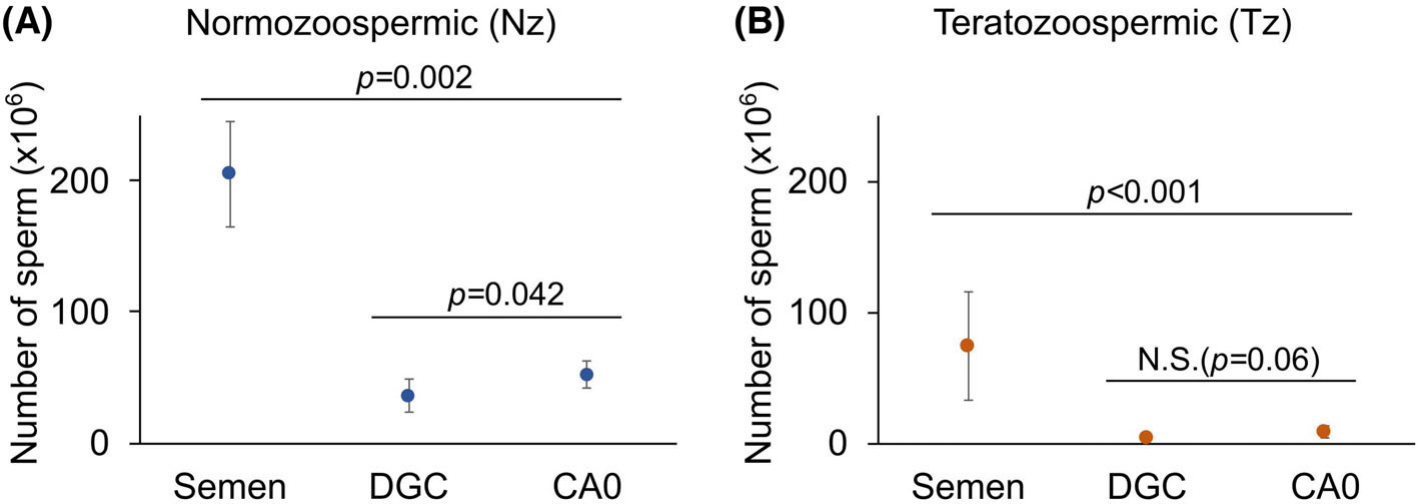
The numbers of cells recovered by density gradient centrifugation (DGC) and by CA0 chambers (CA0) are comparable. (A) Normozoospermic (Nz) samples. Median of cells in semen samples from Nz donors compared with the numbers of sperm recovered by DGC and by CA0; *n* = 12. (B) Teratozoospermic (Tz) samples. Median of cells in semen samples from Tz patients compared with the numbers of sperm recovered by DGC and by CA0 chambers; *n* = 36. Dots represent the median and bars represent the MAD. The statistical test used is Wilcoxon signed rank test for paired data (two‐sided). As indicated in each plot, statistically significant differences were considered when *P* ≤ 0.05, *P* ≤ 0.01, or *P* ≤ 0.001. N.S. = not statistically significant, when *P* ≥ 0.05.

The main aim of this work was to validate the use of CA0 chambers as a suitable sperm selection method to obtain good quality samples for ARTs. We first performed cell counts in Nz and Tz semen samples; as expected, cell counts in Tz samples were lower than in Nz samples (median sperm counts: 205 × 10^6^ for Nz semen; 74.5 × 10^6^ for Tz semen). We then separated equal volumes of each sample using either DGC or CA0 chambers and compared recovery values. We obtained a higher sperm count in Nz compared to Tz samples. Median sperm counts for Nz samples were as follows: 36 × 10^6^ for DGC; 52 × 10^6^ for CA0 (Fig. 4A, Table S1). In the case of Tz samples, median sperm counts were: 4.8 × 10^6^ for DGC; 9.2 × 10^6^ for CA0 (Fig. 4B, Table S1). There were only significant differences in the recovery values obtained between the two separation methods in the case of Nz samples.

**Fig. 5:**
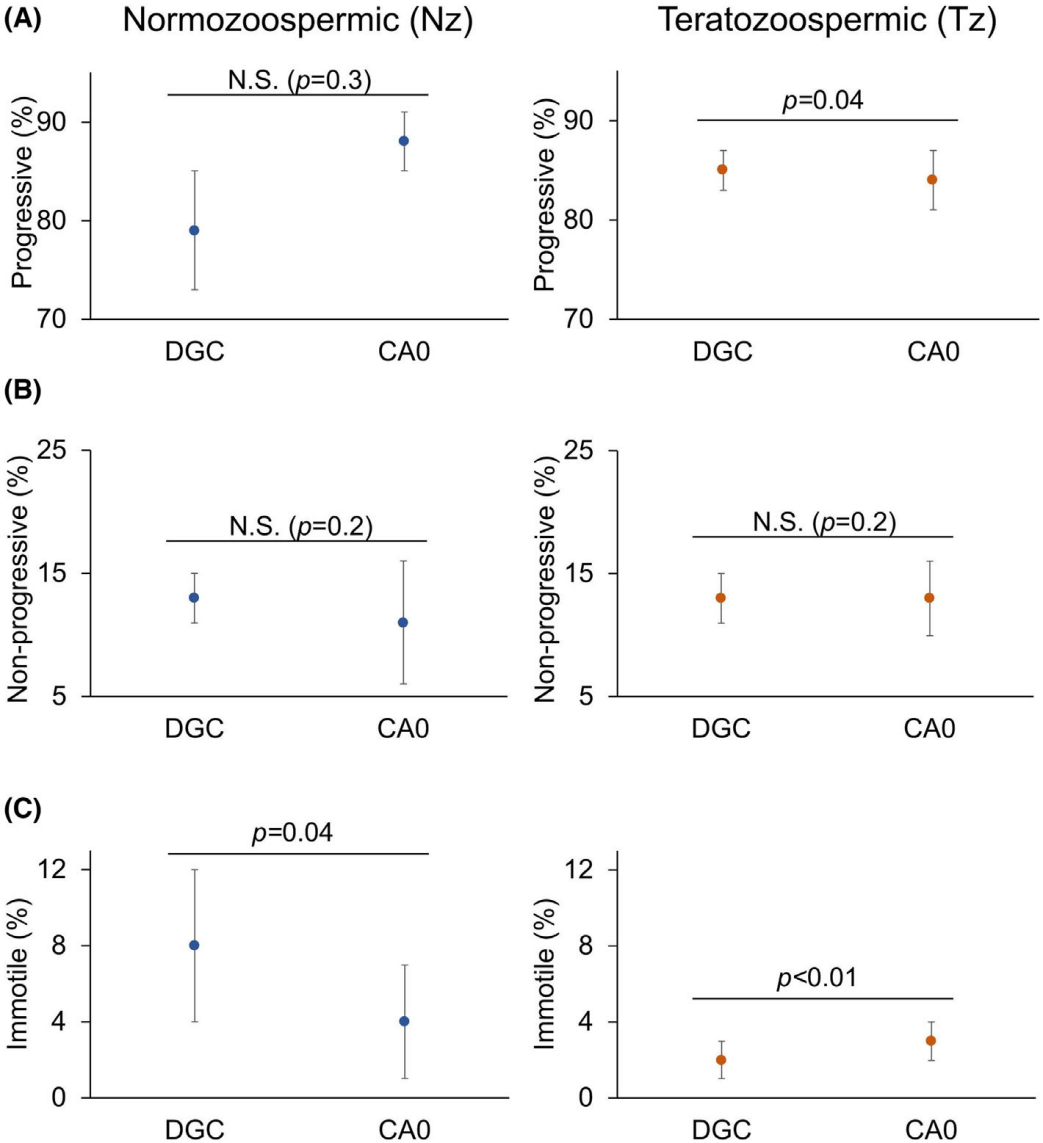
The motility types of sperm recovered by density gradient centrifugation (DGC) and by CA0 chambers (CA0) are comparable. The percentages of progressive (A), non‐progressive (B), and immotile (C) sperm in Nz (left; *n* = 9) and Tz samples (right; *n* = 46) are shown. Dots represent the median and bars represent the MAD. The statistical test used is Wilcoxon signed rank test for paired data (two‐sided). As indicated in each plot, statistically significant differences were considered when *P* ≤ 0.05, *P* ≤ 0.01, or *P* ≤ 0.001. N.S. = not statistically significant, when *P* ≥ 0.05.

To compare sperm motility after separation with the two methods, we analyzed and classified the cells into three categories: progressive, non‐progressive, and immotile. As shown in Fig. 5, there were no significant differences between the non‐progressive motility types observed for Nz and Tz samples separated using either DGC or CA0 chambers. We observed a slightly higher progressive motility percentage in Tz samples when DGC was used. In the case of immotile sperm percentages, the values were lower for Nz samples when CA0 was used; conversely, the values were higher for Tz samples when CA0 was employed. The cell percentage values in each motility category were as follows: for Nz samples: 79 ± 6 progressive, 13 ± 2 non‐progressive, and 8 ± 4 immotile for DGC; and 88 ± 3 progressive, 11 ± 5 non‐progressive, and 4 ± 3 immotile for CA0 (Fig. 5A–C left; Table S1). For Tz samples: 85 ± 2 progressive, 13 ± 2 non‐progressive, and 2 ± 1 immotile for DGC; and 84 ± 3 progressive, 13 ± 3 non‐progressive, and 3 ± 1 immotile for CA0 (Fig. 5A–C right; Table S1).

While the quality of sperm samples is usually assessed by merely evaluating cell counts, morphology, DNA integrity, and motility types, in this work we were interested in further validating the separation method using CA0 chambers. Previously Hsu et al. performed DNA integrity assays comparing three sperm separation methods: DGC, Zymot (sperm separation device which sorts sperm within a space‐constrained microfluidic sorting chip, retrieving processed samples from the outlet port by using a syringe) and CA0. Only CA0 and Zymot had consistent efficiency in eliminating DNA damaged spermatozoa in normozoospermic and non‐normozoospermic donors. By contrast, DGC induces oxidative stress and the subsequent increase in sperm DNA fragmentation index [31]. Therefore, in this work we focused on two key molecular and functional parameters, namely Em resting value and the [Ca^2+^]_i_ response to P4.

**Fig. 6:**
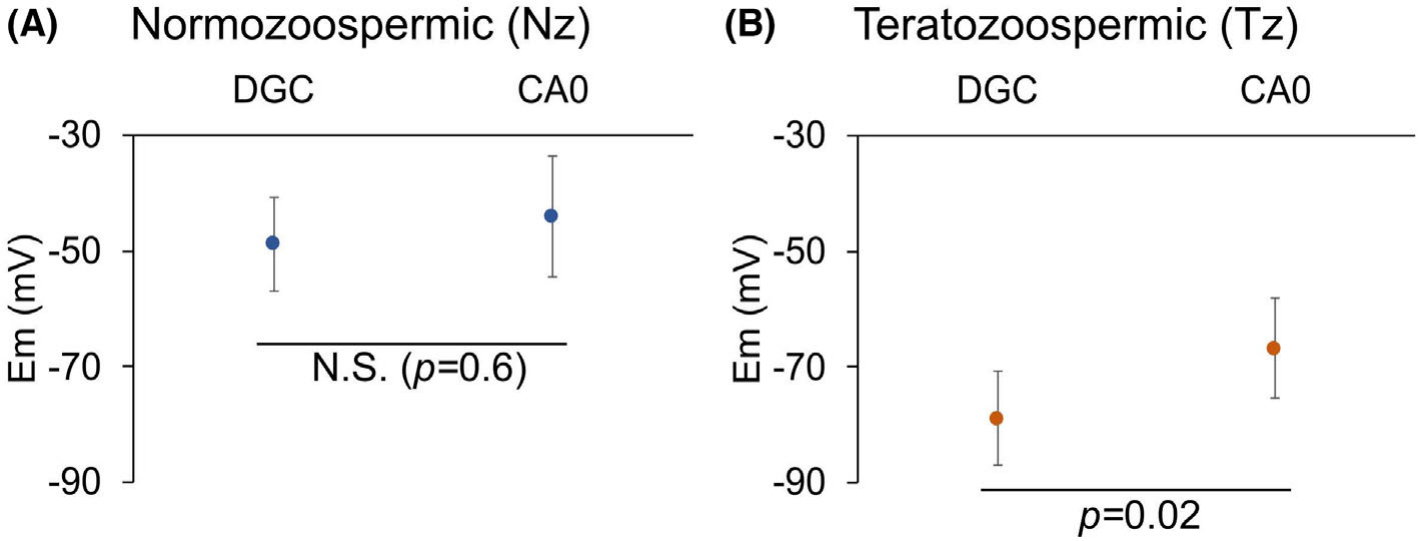
Sperm from Tz patients, but not from Nz donors, show a more hyperpolarized Em when separated using density gradient centrifugation (DGC) compared to those separated using CA0 chambers (CA0). (A) Comparison of resting Em values of sperm from Nz donors (*n* = 11) separated using DGC versus CA0. (B) Comparison of resting Em values of sperm from Tz patients (*n* = 22) separated using DGC versus CA0. The value shown in dots corresponds to the median Em value in mV. Bars represent the MAD. The statistical test used is Wilcoxon signed rank test for paired data (two‐sided). As indicated in each plot, statistically significant differences were considered when *P* ≤ 0.05, *P* ≤ 0.01, or *P* ≤ 0.001. N.S. = not statistically significant, when *P* ≥ 0.05.

The resting Em values obtained in Nz samples separated by DGC (−48.8 ± 8.1 mV) or CA0 chambers (−44 ± 10.4 mV) were similar (Fig. 6A, Table S2). In contrast, for Tz samples we found that sperm separated by DGC exhibited slightly more hyperpolarized Em resting values (−78.9 ± 8.1 mV) than those selected using CA0 chambers (−66.8 ± 8.6 mV) (Fig. 6B, Table S2).

**Fig. 7:**
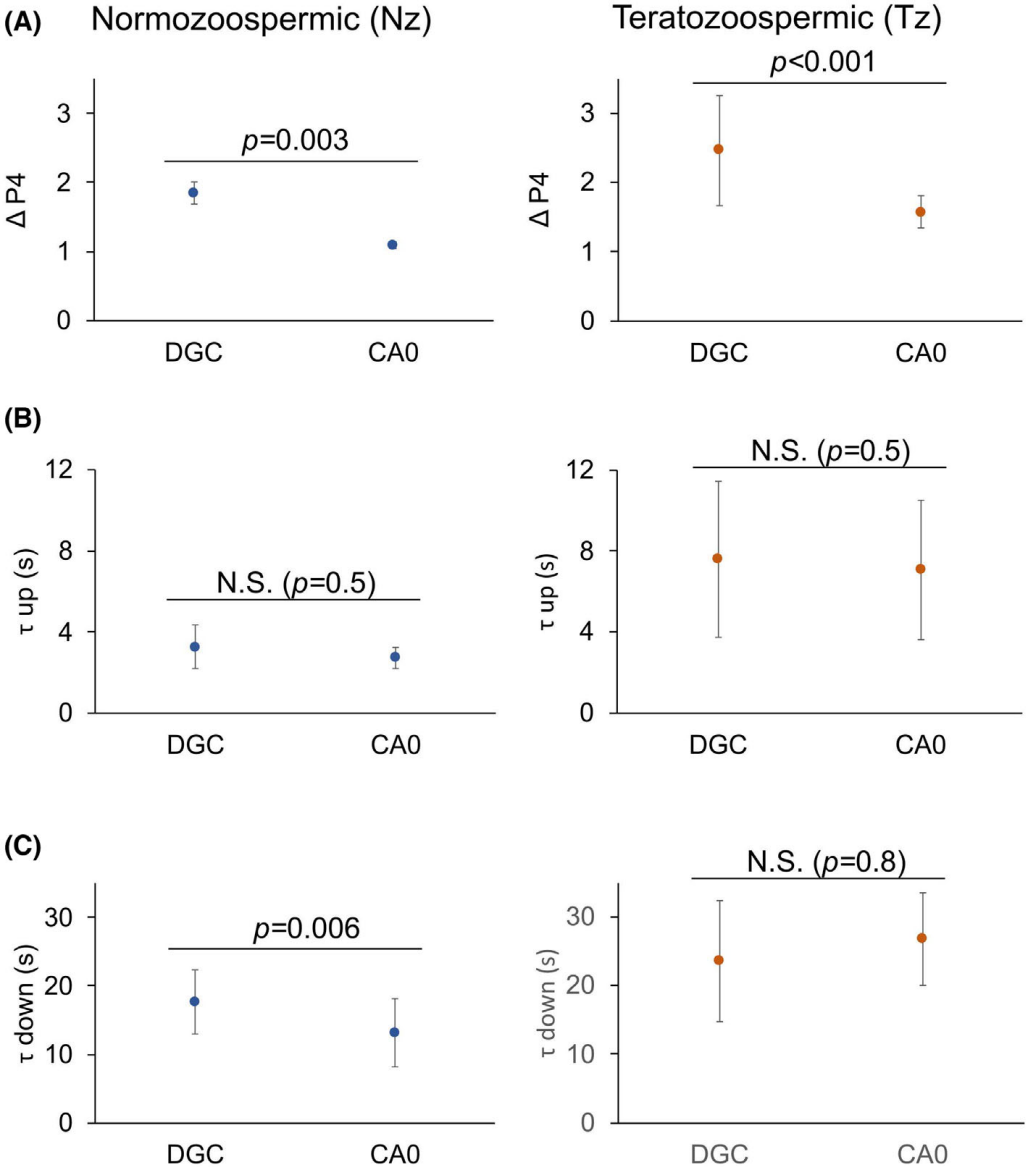
Intracellular Ca^2+^ measurements kinetics are overall comparable in sperm separated using density gradient centrifugation (DGC) and those separated using CA0 chambers (CA0). The median ΔP4 (A), τ up (B), and τ down (C) values for Nz (left; *n* = 9) and Tz (right; *n* = 46) sperm samples are shown. The dots represent the median and bars represent the MAD. The statistical test used is the Wilcoxon signed rank test for paired data (two‐sided). As indicated in each plot, statistically significant differences were considered when *P* ≤ 0.05, *P* ≤ 0.01, or *P* ≤ 0.001. N.S. = not statistically significant, when *P* ≥ 0.05.

In order to compare the P4‐induced [Ca^2+^]_i_ increase, we evaluated the following kinetic parameters of the transient response (Fig. 3A): ΔP4 (maximal fluorescence response – basal fluorescence) (Fig. 7A); the half‐time to achieve the maximum response (τ up; Fig. 7B); and the half‐time to return to basal levels after the stimulus (τ down; Fig. 7C). In Nz samples, we found a significant difference in ΔP4, being greater in cells obtained with DGC (1.9 ± 0.2) than in those from CA0 chambers (1.1 ± 0.1) (Fig. 7A left, Table S3). In contrast, no significant differences were found between the two separation methods when τ up values were compared (3.3 ± 1.1 s for DGC; 2.7 ± 0.5 s for CA0). However, we detected higher τ down values in cells obtained with DGC (17.7 ± 4.7 s for DGC; 13.2 ± 4.9 s for CA0) (Fig. 7B,C left; Table S3). In Tz samples, we found that ΔP4 was also slightly greater (2.5 ± 0.1) for DGC samples compared to those obtained with CA0 chambers (1.6 ± 0.2) (Fig. 7A right, Table S3). Similar to Nz samples, in Tz samples there were no significant differences in τ up values (7.6 ± 3.9 s for DGC; 7 ± 3.5 s for CA0) and τ down values (23.5 ± 8.8 s for DGC; 26.7 ± 6.7 s for CA0) between separation methods (Fig. 7B,C, right; Table S3).

**Fig. 8:**
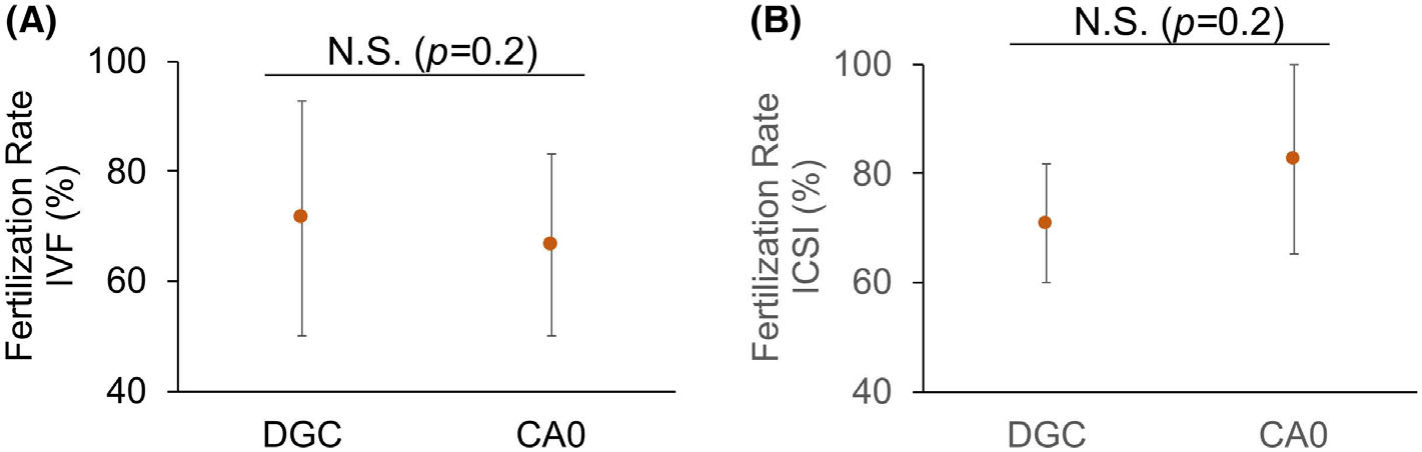
Sperm from Tz patients separated using centrifugation density (DGC) gradient and using CA0 chambers (CA0) yield equivalent fertilization rates both through IVF and ICSI. (A) Median fertilization rates (%) for IVF (*n* = 72) with sperm separated using DGC versus CA0. (B) Median percentage fertilization rates (%) for ICSI (*n* = 22) with sperm separated using DGC versus CA0. Dots represent the median and bars represent the MAD. The statistical test used is Wilcoxon signed rank test for paired data (two‐sided). As indicated in each plot, statistically significant differences were considered when *P* ≤ 0.05, *P* ≤ 0.01 or *P* ≤ 0.001. N.S. = not statistically significant, when *P* ≥ 0.05.

Finally, since Tz samples were obtained from patients undergoing ART treatment at CITMER, we were able to compare the fertilization efficiency between DGC and CA0 samples in both IVF (Fig. 8A; Table S4) and ICSI (Fig. 8B; Table S4). There were no significant differences observed for either ART, with fertilization rates for IVF measuring at 71.4 ± 21.4% with sperm separated by DGC and at 66.6 ± 16.6% with sperm from CA0 chambers, and at 70.1 ± 10.8% and 82.7 ± 17.4%, respectively, in the case of ICSI.

## Discussion

Considering that most of the male patients who attend fertility clinics suffer from low sperm count, sperm selection strategies may need to be adjusted according to the patient's individual condition. At the same time, the procedures should ideally avoid cell damage, as well as morphological and functional alterations; moreover, they should be efficient in removing dead cells and, when present, other undesirable cells, such as leukocytes and bacteria.

Typically, comparative studies of sperm selection methods have assessed parameters limited to morphology, motility, DNA integrity, and in some cases the levels of reactive oxygen species (ROS). Few studies have focused on molecular and functional factors such as Ca^2+^ homeostasis and Em values, despite the fact that both of them have been proposed as predictors for ART success [15, 33, 34, 35, 36, 37]. Given that the goal of sperm selection is fertilization success, it is of paramount importance to evaluate molecular and functional parameters, along with fertilization rates, when validating a new separation strategy.

It is important to mention that the sperm separation procedure can have an impact on Ca^2+^ homeostasis. For example, samples obtained by DGC have been shown to have a higher frequency of spontaneous Ca^2+^ oscillations, compared to sperm obtained by swim‐up [15]. Additionally, Kelly *et al*. reported that upon P4 stimulation, the maximal [Ca^2+^]_i_ response is lower and the [Ca^2+^]_i_ oscillation frequency is higher in sperm from subfertile patients compared to Nz donors [33]. Independent groups have also reported that sperm from subfertile patients have a more depolarized Em than those from Nz donors [35, 36].

Some studies have analyzed fertilization rates when comparing sperm selection methods. In one of these studies, the use of microfluidic chambers resulted in the enrichment of sperm with high motility, good morphology, and a significant reduction in the percentage of sperm with DNA damage [42]. The use of such chambers provides additional advantages, including the possibility of incorporating devices to maintain physiological temperature or to produce a negative flow (which may promote rheotaxis), and even the option of placing oocytes at one end of the chamber to perform IVF *in situ*, thus reducing oocyte damage due to manipulation. Additionally, oocyte development can be monitored directly in the microfluidic chamber. However, the use of these chambers has so far been limited to research purposes, as they have not yet been tested in fertility clinics.

In the present work, we used Nz and Tz samples to evaluate a novel and easy‐to‐use sperm separation device (the CA0 chamber), by analyzing cell recovery and motility, resting Em, and P4‐induced [Ca^2+^]_i_ responsiveness, along with fertilization rates employing ICSI and/or IVF. We compared sperm selected with CA0 chambers against samples obtained through DGC, the latter being the method most frequently used in fertility clinics in Mexico.

In terms of cell recovery, the results were comparable both for Nz and Tz samples, with the median value being slightly higher in Nz samples for CA0 compared to DGC. Overall, the number of cells recovered with both separation methods was comparable to those previously reported [31]. In relation to motility profiles, Nz samples displayed comparable percentage values, except for a slightly lower percentage of immotile sperm when CA0 was used. In the case of Tz samples, when CA0 was used, the percentage of cells with progressive motility was marginally lower, while the percentage of immotile sperm was slightly higher; there was no difference in the percentage of sperm with non‐progressive motility between the two separation methods. The values we obtained for progressive (> 75%), non‐progressive (< 20%), and immotile (< 10%) sperm are similar to those from previous studies using either the same DGC gradient protocol [15, 21] or a different one [10]. Recently, Hsu and colleagues reported a comparison of several motility parameters from Nz and non‐Nz sperm samples selected using either DGC, Zymot devices, and CA0 chambers. The motility profiles they observed are somewhat different from ours; the discrepancies may be in part due to the fact that we used different incubation temperatures and times, and smaller sample sizes [31]. In terms of cell recovery, DNA fragmentation, and cell morphology, Hsu and collaborators found that CA0 provided better results than DGC. In this study, we explored additional functional parameters that complement the data from Hsu and collaborators.

A hyperpolarized resting Em has been correlated positively to fertilization success [35, 36]. We only found significant differences in Em values of sperm from Tz patients, with cells separated by DGC having a more hyperpolarized Em value compared to sperm obtained with CA0. This may be due to the effect of centrifugation, since previous reports indicate that sperm obtained by DGC show higher levels of tyrosine phosphorylation, hyperactivation of motility, and higher [Ca^2+^]_i_ oscillations, all of which are capacitation markers [15]. Accordingly, the Em hyperpolarization we observed could presumably be a reflection of a higher capacitated state. However, the higher hyperpolarized Em in Tz patients had no significant effect on fertilization rates, as these were similar using sperm obtained through DGC and CA0, both in IVF and ICSI procedures.

As for [Ca^2+^]_i_ measurements, previous reports have considered only the magnitude of the P4‐induced [Ca^2+^]_i_ increase, which correlates positively with fertilization success [33]. We also calculated the τ up and τ down of the response, as it provides information as to how fast the cell can respond and recover from the stimulus. Our data indicate that the [Ca^2+^]_i_ kinetics observed upon P4 addition are similar when both sperm separation methods were used.

Finally, we evaluated the fertilization rates of sperm separated with either DGC or CA0, using the two most commonly employed ART procedures in Mexico, namely IVF and ICSI. We did not find a significant difference in the fertilization rates between the two sperm separation methods, either through IVF or ICSI.

The CA0 device, a noninvasive sperm selection method, offers potential advantages for patients with male infertility factors. By selecting for sperm with adequate motility and minimal DNA fragmentation, fertilization rates may be improved while reducing the risk of embryo aneuploidy. This is particularly beneficial for men with low sperm counts, poor motility, or high levels of DNA damage, as it can enhance the quality of sperm used in assisted reproductive technologies (ART) such as intrauterine insemination (IUI) and *in vitro* fertilization (IVF) [33]. However, the clinical data on the CA0 device is still limited, and its effectiveness in various patient populations remains to be fully established. Further research is needed to determine its optimal use and potential long‐term outcomes for different infertility scenarios.

Additionally, it is important to emphasize that the CA0 device offers potential advantages over density gradient centrifugation by being noninvasive, selecting sperm with adequate motility, and potentially reducing DNA fragmentation. However, it is a newer technology with limited clinical data and may be more expensive and less readily available. Density gradient centrifugation, while well‐established and widely accessible, can be damaging to sperm and may not effectively select for the most motile sperm or significantly reduce DNA fragmentation.

## Conclusions

Our results indicate that overall, non‐Nz sperm selected using CA0 chambers have comparable functional parameters to those separated with DGC, which is currently one of the most widely used sperm separation procedures in fertility clinics. These results are further supported by the fact that we observed no significant differences in fertilization rates between the two sperm separation procedures, whether ART or ICSI was employed. To our knowledge, this is the first report of a study being conducted to validate the use of CA0 chambers as a suitable alternative to sperm separation. Due to their ease of use, the personnel processing the sample with CA0 chambers do not require prior training and skilled experience. Furthermore, sperm selection with CA0 chambers is a more affordable method than DGC, as less disposable/sterile materials are employed, and it does not require additional specialized laboratory equipment such as a centrifuge, which is costly to maintain. As additional advantages, sperm selection with CA0 chambers is faster and involves fewer steps, in turn reducing sample manipulation and processing times.

## Conflict of interest

The authors declare no conflict of interest.

## Peer review

The peer review history for this article is available at https://www.webofscience.com/api/gateway/wos/peer‐review/10.1002/2211‐5463.70073.

## Author contributions

CT and IM conceived and designed the project. JCC, PT, GC‐M, MGF‐M, DF and LV acquired, analyzed, and interpreted the data. JCC, MBT, and CT wrote the paper.

## Supporting information


**Table S1.** Sperm number (×10^6^) from semen samples and after separation. Percentage of motility types such as progressive, non‐progressive and immotile according to WHO manual (WHO, 2010).
**Table S2.** Membrane Potential values (mV) obtained after each separation method.
**Table S3.** Ca^2+^ increase produced by addition of 3 μm P4 (Δ value), including half‐time (seconds) to the maximum response (τ up) and half‐time (seconds) to recover to basal levels after the Progesterone stimulus (τ down).
**Table S4.** Percentage of fertilization rate using IVF and ICSI with sperm from teratozoospermic patients.

## Data Availability

All the data reported in this work is included as Tables S1–S4.
