# Supplementary material for: Functional parameters of spermatozoa obtained by a new selection device
Source: FEBS Open Bio. 2025 Jul 14;15(9):1471–84. doi: 10.1002/2211-5463.70073 (PMC12401173; doi:10.1002/2211-5463.70073)
Supplement: Supplementary file 1 — Table S1. Sperm number (×106) from semen samples and after separation. Percentage of motility types such as progressive, non‐progressive and immotile according to WHO manual (WHO, 2010). Table S2. Membrane Potential values (mV) obtained after each separation method. Table S3. Ca2+ increase produced by addition of 3 μm P4 (Δ value), including half‐time (seconds) to the maximum response (τ up) and half‐time (seconds) to recover to basal levels after the Progesterone stimulus (τ down). Table S4. Percentage of fertilization rate using IVF and ICSI with sperm from teratozoospermic patients. [file FEB4-15-1471-s001.docx]

**Table S1.** Sperm number (X 10^6^) from semen samples and after separation. Percentage of motility types such as progressive, non- progressive and immotile according to WHO manual (WHO, 2010).

| **Normozoospermic** | | | | | | | | | |
| --- | --- | --- | --- | --- | --- | --- | --- | --- | --- |
|  | Sperm concentration (X 10^6^) | | | Motility type | | | | | |
|  |  | | | Progressive | | Non-Progressive | | Immotile | |
| Donor | Semen | DGC | CA0 | DGC | CA0 | DGC | CA0 | DGC | CA0 |
| 1 | 210 | 70 | 50 | 86 | 85 | 11 | 11 | 3 | 4 |
| 2 | 170 | 50 | 40 | 73 | 90 | 16 | 5 | 11 | 5 |
| 3 | 65 | 25 | 20 | 79 | 95 | 13 | 4 | 8 | 1 |
| 4 | 200 | 45 | 60 | 82 | 88 | 11 | 11 | 7 | 1 |
| 5 | 175 | 18 | 15 | 82 | 93 | 11 | 6 | 7 | 1 |
| 6 | 210 | 55 | 40 | 51 | 90 | 15 | 6 | 34 | 4 |
| 7 | 135 | 40 | 35 | 67 | 41 | 17 | 20 | 16 | 20 |
| 8 | 300 | 75 | 55 | 66 | 63 | 15 | 22 | 19 | 15 |
| 9 | 150 | 25 | 35 | 83 | 82 | 13 | 14 | 4 | 4 |
| 10 | 250 | 60 | 50 |  |  |  |  |  |  |
| 11 | 340 | 60 | 35 |  |  |  |  |  |  |
| 12 | 240 | 40 | 30 |  |  |  |  |  |  |
| **Median** | **205** | **36** | **52** | **79** | **88** | **13** | **11** | **8** | **4** |
| **MAD** | **40** | **12.5** | **10** | **6** | **3** | **2** | **5** | **4** | **3** |
| **Teratozoospermic** | | | | | | | | | |
|  | Sperm concentration (X 10^6^) | | | Motility type | | | | | |
|  |  |  |  | Progressive | | Non-Progressive | | Immotile | |
| Donor | Semen | DGC | CA0 | DGC | CA0 | DGC | CA0 | DGC | CA0 |
| 1 | 30 | 4.1 | 5.2 | 92 | 77 | 7 | 17 | 1 | 6 |
| 2 | 30 | 4.1 | 5.2 | 92 | 77 | 7 | 17 | 1 | 6 |
| 3 | 33 | 3.8 | 3.3 | 90 | 91 | 9 | 8 | 1 | 1 |
| 4 | 33 | 3.8 | 3.3 | 90 | 91 | 9 | 8 | 1 | 1 |
| 5 | 105 | 4.8 | 3.9 | 90 | 85 | 9 | 13 | 1 | 2 |
| 6 | 105 | 4.8 | 3.9 | 90 | 85 | 9 | 13 | 1 | 2 |
| 7 | 145 | 17 | 14 | 85 | 82 | 13 | 15 | 2 | 3 |
| 8 | 145 | 17 | 14 | 85 | 82 | 13 | 15 | 2 | 3 |
| 9 | 56 | 11 | 5 | 89 | 91 | 10 | 9 | 1 | 0 |
| 10 | 56 | 11 | 5 | 89 | 91 | 10 | 9 | 1 | 0 |
| 11 | 30 | 2.8 | 12 | 94 | 85 | 5 | 13 | 1 | 2 |
| 12 | 30 | 2.8 | 12 | 94 | 85 | 5 | 13 | 1 | 2 |
| 13 | 34 | 4.8 | 5 | 89 | 96 | 10 | 2 | 1 | 2 |
| 14 | 34 | 4.8 | 5 | 89 | 96 | 10 | 2 | 1 | 2 |
| 15 | 128 | 48 | 5.4 | 80 | 95 | 17 | 2 | 3 | 3 |
| 16 | 128 | 48 | 5.4 | 80 | 95 | 17 | 2 | 3 | 3 |
| 17 | 228 | 3.4 | 30 | 86 | 96 | 12 | 4 | 2 | 0 |
| 18 | 190 | 3.4 | 30 | 86 | 96 | 12 | 4 | 2 | 0 |
| 19 | 57 | 5 | 10 | 83 | 80 | 15 | 18 | 2 | 2 |
| 20 | 57 | 5 | 10 | 83 | 80 | 15 | 18 | 2 | 2 |
| 21 | 55 | 4.4 | 5.7 | 86 | 85 | 11 | 13 | 3 | 2 |
| 22 | 55 | 4.4 | 5.7 | 86 | 85 | 11 | 13 | 3 | 2 |
| 23 | 58 | 3 | 16 | 84 | 83 | 14 | 14 | 2 | 3 |
| 24 | 58 | 3 | 16 | 84 | 83 | 14 | 14 | 2 | 3 |
| 25 | 48 | 4.3 | 2.8 | 89 | 86 | 9 | 10 | 2 | 4 |
| 26 | 48 | 4.3 | 2.8 | 89 | 86 | 9 | 10 | 2 | 4 |
| 27 | 125 | 4.9 | 20 | 85 | 82 | 13 | 15 | 2 | 3 |
| 28 | 125 | 4.9 | 20 | 85 | 82 | 13 | 15 | 2 | 3 |
| 29 | 150 | 3.7 | 8.3 | 86 | 79 | 12 | 17 | 2 | 4 |
| 30 | 150 | 3.7 | 8.3 | 86 | 79 | 12 | 17 | 2 | 4 |
| 31 | 133 | 5.3 | 10.1 | 84 | 77 | 15 | 19 | 1 | 4 |
| 32 | 133 | 5.3 | 10.1 | 84 | 77 | 15 | 19 | 1 | 4 |
| 33 | 91 | 5.3 | 10.3 | 82 | 83 | 16 | 12 | 2 | 5 |
| 34 | 91 | 5.3 | 10.3 | 82 | 83 | 16 | 12 | 2 | 5 |
| 35 | 104 | 7.9 | 20 | 84 | 83 | 14 | 16 | 2 | 1 |
| 36 | 104 | 7.9 | 20 | 84 | 83 | 14 | 16 | 2 | 1 |
| 37 |  |  |  | 85 | 84 | 13 | 13 | 2 | 3 |
| 38 |  |  |  | 85 | 84 | 13 | 13 | 2 | 3 |
| 39 |  |  |  | 85 | 87 | 14 | 12 | 1 | 1 |
| 40 |  |  |  | 85 | 87 | 14 | 12 | 1 | 1 |
| 41 |  |  |  | 80 | 77 | 17 | 21 | 3 | 2 |
| 42 |  |  |  | 80 | 77 | 17 | 21 | 3 | 2 |
| 43 |  |  |  | 83 | 78 | 15 | 18 | 2 | 4 |
| 44 |  |  |  | 83 | 78 | 15 | 18 | 2 | 4 |
| 45 |  |  |  | 86 | 87 | 13 | 10 | 1 | 3 |
| 46 |  |  |  | 86 | 87 | 13 | 10 | 1 | 3 |
| **Median** | **74.5** | **4.8** | **9.2** | **85** | **84** | **13** | **13** | **2** | **3** |
| **MAD** | **41** | **0.9** | **4.2** | **2** | **3** | **2** | **3** | **1** | **1** |

**Table S2.** Membrane Potential values (mV) obtained after each separation method.

| **Normozoospermic** | | |
| --- | --- | --- |
| Donor | DGC | CA0 |
| 1 | -43 | -32.5 |
| 2 | -49 | -66.8 |
| 3 | -57.5 | -49 |
| 4 | -74.2 | -38.5 |
| 5 | -38.9 | -32.6 |
| 6 | -48.8 | -42.3 |
| 7 | -56.9 | -68 |
| 8 | -44.4 | -44 |
| 9 | -38.1 | -37.1 |
| 10 | -58.4 | -54.4 |
| 11 | -44.5 | -61.6 |
| **Median** | **-48.8** | **-44** |
| **MAD** | **8.1** | **10.4** |

| **Teratozoospermic** | | |
| --- | --- | --- |
| Donor | DGC | CA0 |
| 1 | -77.8 | -65 |
| 2 | -92.6 | -74 |
| 3 | -63.9 | -12.3 |
| 4 | -73.4 | -67.8 |
| 5 | -83.9 | -79.7 |
| 6 | -86.3 | -68.7 |
| 7 | -95.7 | -61.6 |
| 8 | -99.1 | -75.1 |
| 9 | -98.3 | -75.7 |
| 10 | -100.6 | -100.9 |
| 11 | -79.4 | -80.9 |
| 12 | -80.7 | -90 |
| 13 | -72.4 | -64.4 |
| 14 | -78.4 | -38.4 |
| 15 | -57.6 | -65.8 |
| 16 | -85.6 | -85.6 |
| 17 | -59.9 | -57.4 |
| 18 | -47.9 | -52.9 |
| 19 | -70.4 | -63.6 |
| 20 | -80.6 | -75.1 |
| 21 | -71.3 | -29.3 |
| 22 | -42.3 | -64.8 |
| **Median** | **-78.9** | **-66.8** |
| **MAD** | **8.1** | **8.6** |

**Table S3.** Ca^2+^ increase produced by addition of 3 µM Pg (Δ value), including half-time (seconds) to the maximum response (τ up) and half-time (seconds) to recover to basal levels after the Pg stimulus (τ down).

| **Normozoospermic** | | | | | | |
| --- | --- | --- | --- | --- | --- | --- |
|  | Response to 3 µM Pg (Δ value) | | τ up | | τ down | |
| Donor | DGC | CA0 | DGC | CA0 | DGC | CA0 |
| 1 | 1.47 | 1.42 | 4.47 | 2.39 | 42.8 | 40.2 |
| 2 | 1.94 | 1.18 | 2.89 | 2.96 | 38.0 | 16.9 |
| 3 | 1.67 | 1.14 | 2.02 | 3.95 | 21.4 | 16.0 |
| 4 | 1.85 | 1.18 | 0.91 | 2.96 | 13.4 | 16.9 |
| 5 | 2.21 | 1.11 | 2.01 | 4.91 | 17.7 | 6.6 |
| 6 | 1.92 | 1.07 | 2.19 | 2.38 | 18.6 | 13.2 |
| 7 | 1.89 | 1.05 | 3.27 | 2.09 | 12.3 | 3.8 |
| 8 | 1.41 | 1.02 | 3.26 | 1.47 | 13.1 | 8.6 |
| 9 | 1.69 | 1.02 | 3.83 | 0.74 | 13.1 | 6.5 |
| 10 | 1.11 | 1.09 | 3.35 | 2.73 | 12.6 | 8.3 |
| 11 | 1.96 | 1.07 | 6.07 | 3.25 | 27.8 | 21.3 |
| **Median** | **1.9** | **1.1** | **3.3** | **2.7** | **17.7** | **13.2** |
| **MAD** | **0.2** | **0.1** | **1.1** | **0.5** | **4.7** | **4.9** |
| **Teratozoospermic** | | | | | | |
|  | Response to 3 µM Pg (Δ value) | | τ up | | τ down | |
| Donor | DGC | CA0 | DGC | CA0 | DGC | CA0 |
| 1 | 1.77 | 1.77 | 5.8 | 5.8 | 42.8 | 33.6 |
| 2 | 3.56 | 1.61 | 4.8 | 11.9 | 65.8 | 3.0 |
| 3 | 1.65 | 1.5 | 7.6 | 20.2 | 48.3 | 3.4 |
| 4 | 3.92 | 0.96 | 6.4 | 21.0 | 74.5 | 49.5 |
| 5 | 2.46 | 1.55 | 5.5 | 4.5 | 32.9 | 35.8 |
| 6 | 3.27 | 2.16 | 9.0 | 7.4 | 26.8 | 32.7 |
| 7 | 2.03 | 5.96 | 11.4 | 4.5 | 12.5 | 35.8 |
| 8 | 3.81 | 2.21 | 4.1 | 2.5 | 41.2 | 27.0 |
| 9 | 2.91 | 1.57 | 3.5 | 4.5 | 14.2 | 35.8 |
| 10 | 2.01 | 1.81 | 3.6 | 8.6 | 30.4 | 35.8 |
| 11 | 2.8 | 1.66 | 5.5 | 9.8 | 22.7 | 22.7 |
| 12 | 1.53 | 1.52 | 2.1 | 3.5 | 33.9 | 4.8 |
| 13 | 1.85 | 1.26 | 1.9 | 7.6 | 24.5 | 2.8 |
| 14 | 2.15 | 1.74 | 14.2 | 7.6 | 23.5 | 24.5 |
| 15 | 4.08 | 1.89 | 10.1 | 3.0 | 38.4 | 20.8 |
| 16 | 1.09 | 2.21 | 8.3 | 5.8 | 6.9 | 13.9 |
| 17 | 1.93 | 1.55 | 9.4 | 6.3 | 11.8 | 24.8 |
| 18 | 4.2 | 2.05 | 1.4 | 1.8 | 28.7 | 25.3 |
| 19 | 1.69 | 2.01 | 5.0 | 4.0 | 8.7 | 29.1 |
| 20 | 3.56 | 1.53 | 4.6 | 3.6 | 15.1 | 36.2 |
| 21 | 2.99 | 2.92 | 3.1 | 5.3 | 31.2 | 30.4 |
| 22 | 2.6 | 1.35 | 10.1 | 10.1 | 27.0 | 27.0 |
| 23 | 1.25 | 1.04 | 10.6 | 67.3 | 23.5 | 23.5 |
| 24 | 2.07 | 1.4 | 29.2 | 7.1 | 22.2 | 37.8 |
| 25 | 4.16 | 1.57 | 7.6 | 11.6 | 41.5 | 26.1 |
| 26 | 1.42 | 1.83 | 35.8 | 3.1 | 3.7 | 3.7 |
| 27 | 1.12 | 1.19 | 16.5 | 18.1 | 16.2 | 24.3 |
| 28 | 3.6 | 1.3 | 14.9 | 4.2 | 29.9 | 30.1 |
| 29 | 2.82 | 1.19 | 5.1 | 11.8 | 20.0 | 20.0 |
| 30 | 2.46 | 1.76 | 2.5 | 3.6 | 11.8 | 29.9 |
| 31 | 2.07 | 1.4 | 29.2 | 7.1 | 22.2 | 37.8 |
| 32 | 1.27 | 2.26 | 14.5 | 14.9 | 9.6 | 26.7 |
| 33 | 3.11 | 1.51 | 23.6 | 16.2 | 14.7 | 14.5 |
| 34 | 3.3 | 1.48 | 23.6 | 16.1 | 20.2 | 13.6 |
| 35 | 1.56 | 1.46 | 21.8 | 10.6 | 27.7 | 32.0 |
| **Median** | **2.5** | **1.6** | **7.6** | **7.1** | **23.5** | **26.7** |
| **MAD** | **0.8** | **0.2** | **3.9** | **3.5** | **8.8** | **6.7** |

**Table S4.** Percentage of Fertilization rate using IVF and ICSI with sperm from teratozoospermic patients.

| **Teratozoospermic** | | | | |
| --- | --- | --- | --- | --- |
|  | % IVF | | % ICSI | |
| Donor | DGC | CA0 | DGC | CA0 |
| 1 | 75 | 25 | 77 | 82 |
| 2 | 50 | 100 | 60 | 80 |
| 3 | 70 | 77.7 | 63.1 | 74 |
| 4 | 57.1 | 71.4 | 100 | 100 |
| 5 | 70 | 55.5 | 66.6 | 50 |
| 6 | 66.6 | 66.6 | 33.3 | 50 |
| 7 | 84.6 | 100 | 60 | 83.3 |
| 8 | 60 | 80 | 75 | 33.3 |
| 9 | 100 | 66.6 | 100 | 100 |
| 10 | 20 | 20 | 87.5 | 87.5 |
| 11 | 50 | 62.5 | 100 | 100 |
| 12 | 44.4 | 50 | 66.6 | 100 |
| 13 | 100 | 100 | 22.2 | 100 |
| 14 | 100 | 100 | 66.6 | 63.6 |
| 15 | 50 | 100 | 80 | 80 |
| 16 | 50 | 60 | 50 | 33.3 |
| 17 | 50 | 50 | 50 | 50 |
| 18 | 66.6 | 56 | 100 | 100 |
| 19 | 33.3 | 100 | 80 | 100 |
| 20 | 90 | 60 | 66.66 | 100 |
| 21 | 100 | 100 | 75 | 85.7 |
| 22 | 100 | 100 | 87.5 | 57.1 |
| 23 | 100 | 71.4 |  |  |
| 24 | 50 | 100 |  |  |
| 25 | 50 | 80 |  |  |
| 26 | 87.5 | 77.7 |  |  |
| 27 | 33.3 | 66.6 |  |  |
| 28 | 100 | 33.3 |  |  |
| 29 | 70 | 18.2 |  |  |
| 30 | 66.6 | 66.6 |  |  |
| 31 | 80 | 63.6 |  |  |
| 32 | 86 | 29 |  |  |
| 33 | 91 | 71.4 |  |  |
| 34 | 86 | 66.6 |  |  |
| 35 | 75 | 75 |  |  |
| 36 | 100 | 33.3 |  |  |
| 37 | 100 | 75 |  |  |
| 38 | 57 | 50 |  |  |
| 39 | 30 | 25 |  |  |
| 40 | 25 | 66.6 |  |  |
| 41 | 100 | 25 |  |  |
| 42 | 70 | 10 |  |  |
| 43 | 72.7 | 56 |  |  |
| 44 | 25 | 71.4 |  |  |
| 45 | 25 | 29 |  |  |
| 46 | 100 | 100 |  |  |
| 47 | 50 | 33.3 |  |  |
| 48 | 42.4 | 86.3 |  |  |
| 49 | 50 | 50 |  |  |
| 50 | 71.4 | 85.7 |  |  |
| 51 | 71.4 | 85.7 |  |  |
| 52 | 37.5 | 75 |  |  |
| 53 | 100 | 75 |  |  |
| 54 | 50 | 75 |  |  |
| 55 | 75 | 75 |  |  |
| 56 | 100 | 100 |  |  |
| 57 | 100 | 33 |  |  |
| 58 | 100 | 50 |  |  |
| 59 | 16.7 | 50 |  |  |
| 60 | 20 | 25 |  |  |
| 61 | 85.7 | 100 |  |  |
| 62 | 85.7 | 50 |  |  |
| 63 | 100 | 80 |  |  |
| 64 | 75 | 33.3 |  |  |
| 65 | 75 | 75 |  |  |
| 66 | 50 | 50 |  |  |
| 67 | 100 | 57.1 |  |  |
| 68 | 40 | 50 |  |  |
| 69 | 25 | 80 |  |  |
| 70 | 100 | 20 |  |  |
| 71 | 75 | 75 |  |  |
| 72 | 100 | 25 |  |  |
| **Median** | **71.4** | **66.6** | **70.8** | **82.7** |
| **MAD** | **21.4** | **16.6** | **10.8** | **17.4** |
